# Kali Cyanate: Provers Needed

**Published:** 1888-01

**Authors:** J. D. Tyrrell

**Affiliations:** Toronto, Canada


### KALI CYANATE: PROVERS NEEDED.

*My dear Doctor:*

I would like to call your attention to a remedy that promises to be of great service to us in the treatment of that terrible scourge to humanity, carcinoma. I want your aid in proving the drug and in verifying said proving by clinical evidence; will you help me, so I may have as full a pathogenesis and as many verifications as possible, so I may complete a paper in time for the International Hahnemannian Association next June? I would like a faithful report of all common and peculiar symptoms and order of relief marked in each case treated; also, I would like day-books of provers.

My attention was called to the remedy by my friend, Dr. E. T. Adams, of Toronto, and in error of size of dose I triturated it for him in ratio of one to two hundred, as he said dose was one-two-hundredth of a grain; since then I got some pure from Darmstadt, and run it up on my "Skinner's Fluxion Centesimal Potentizer," and will send you grafts for your own use and provings.

Kali Hydrocyanate, or, more properly, Cyanate, is the remedy referred to, and must not be confounded with the familiar Cyanide, as it differs from it both chemically, physically, and therapeutically—the chemical formula for Cyanyde you all know, K C N, while the Cyanate is K C N O.

The following is from Petroz' *Collected Writings:*

"In 1829 a woman, living in the Rue St. Nicolas, came to ask my advice about a disease of the tongue, for which she had been under the care of Dr. L'Herminier. The organ was profoundly altered by ulcer, which appeared to me to be cancerous, and which occupied its right side; the edges, especially posteriorly, were indurated, raised, and knotty; speech was difficult, indistinct, and accompanied with much pain. The patient could only take liquid nourishment. Distrusting my own diagnosis, I sent her to Professor Marjolin. She brought back to me the following judgment: 'Cancerous ulcer; no chance of cure but from operation, and this is impossible, for the base of the tongue is involved.'

"In the presence of so grave a disease I turned my thoughts to diminish her sufferings. I prescribed the one-hundredth of a grain of Hydrocyanate of Potassa, to be repeated every fourth day. After fifteen days I again saw the patient. She suffered less, the tongue appeared to me not so thick, the edges less hard, the speech easier. The medicine was continued in the same way. Fifteen days later the patient, whose countenance had lost its gray hue and drawn features, said to me, with joy: 'I begin to be able to eat a crumb of bread.' The Hydrocyanate was continued for a month longer, when the cure was complete. It is now eighteen years ago, and there has been no relapse."

The following is a case treated by my fr·end, Dr. E. T. Adams:

J. S., æt. fifty-eight, hard case and thoroughly whisky-soaked, had been under treatment of many old school physicians —latterly under a well-known surgeon—and each diagnosed cancerous ulcer; prognosis, death. The description of above case exactly gives his condition—a deep ulcer in right side of tongue, in which the first joint of a man's thumb might be laid. Could not take solid food, and only with great pain liquid; was so weak he could scarcely move from his bed. Under the Cyanate he improved quickly, so much so, that this eminent surgeon gave hope of recovery, not knowing a heretical homœopath was attending him. In about eight days was so much stronger he could go for a long walk before six A. M.; the last Dr. Adams saw him he was eating dry bread and boiled beef with comparative ease and comfort. This good surgeon, this "humane aggressor" rescued him from the heretics, and, working upon his ignorance and fears, gained his unwilling consent to have tongue removed. This was done at once, and he lived only about seventeen days, dying in great agony. So much for "scientific" treatment.

The Cyanate of Potassa deserved all the credit for improvement in this case, and I am morally sure it would have cured if left alone.

My own experience has been similar until the one or two cases I had fell into the hands of the Philistines. One lady we know of is so sensitive that any dose either aggravates her condition, or reproduces the chronic sore throat. There is no getting away from the trnth of these facts, and it behooves us to prove this drug and verify the pathogenesis.

Fraternally yours,

J. D. TYRRELL, Toronto, Canada.

